# Discovery of Novel Saponins from the Viscera of the Sea Cucumber *Holothuria lessoni*

**DOI:** 10.3390/md12052633

**Published:** 2014-05-09

**Authors:** Yadollah Bahrami, Wei Zhang, Chris Franco

**Affiliations:** 1 Department of Medical Biotechnology, School of Medicine, Flinders University, Adelaide 5001, SA 5042, Australia; E-Mails: yadollah.bahrami@flinders.edu.au (Y.B.); wei.zhang@flinders.edu.au (W.Z.); 2 Centre for Marine Bioproducts Development, Flinders University, Adelaide 5001, SA 5042, Australia; 3 Australian Seafood Cooperative Research Centre, Mark Oliphant Building, Science Park, Adelaide 5001, SA 5042, Australia; 4 Medical Biology Research Center, Kermanshah University of Medical Sciences, Kermanshah 6714415185, Iran; E-Mail: ybahrami@mbrc.ac.ir

**Keywords:** sea cucumber viscera, saponins, *Holothuria lessoni*, bioactive compounds, MALDI, mass spectrometry, ESI, HPCPC, triterpene glycosides, structure elucidation, marine invertebrate, *Echinodermata*, holothurian

## Abstract

Sea cucumbers, sometimes referred to as marine ginseng, produce numerous compounds with diverse functions and are potential sources of active ingredients for agricultural, nutraceutical, pharmaceutical and cosmeceutical products. We examined the viscera of an Australian sea cucumber *Holothuria lessoni* Massin *et al.* 2009, for novel bioactive compounds, with an emphasis on the triterpene glycosides, saponins. The viscera were extracted with 70% ethanol, and this extract was purified by a liquid-liquid partition process and column chromatography, followed by isobutanol extraction. The isobutanol saponin-enriched mixture was further purified by high performance centrifugal partition chromatography (HPCPC) with high purity and recovery. The resultant purified polar samples were analyzed using matrix-assisted laser desorption/ionization mass spectrometry (MALDI-MS)/MS and electrospray ionization mass spectrometry (ESI-MS)/MS to identify saponins and characterize their molecular structures. As a result, at least 39 new saponins were identified in the viscera of *H*. *lessoni* with a high structural diversity, and another 36 reported triterpene glycosides, containing different aglycones and sugar moieties. Viscera samples have provided a higher diversity and yield of compounds than observed from the body wall. The high structural diversity and novelty of saponins from *H*. *lessoni* with potential functional activities presents a great opportunity to exploit their applications for industrial, agricultural and pharmaceutical use.

## 1. Introduction

Holothurians are sedentary marine invertebrates, commonly known as sea cucumbers, trepang, bêche-de-mer, or gamat [1,2], belonging to the class Holothuroidea of the *Echinodermata* phylum. Sea cucumbers produce numerous compounds with diverse functions and are potential sources of agricultural or agrochemical, nutraceutical, pharmaceutical and cosmeceutical products [3,4,5]. It is for this reason they are called “marine ginseng” in Mandarin.

Even though sea cucumbers contain different types of natural compounds, saponins are their most important and abundant secondary metabolites [6,7,8,9,10,11,12]. Saponins are reported as the major bioactive compound in many effective traditional Chinese and Indian herbal medicines.

Sea cucumber saponins are known to have a wide range of medicinal properties due to their cardiovascular, immunomodulator, cytotoxic, anti-asthma, anti-eczema, anti-inflammatory, anti-arthritis, anti-oxidant, anti-diabetics, anti-bacterial, anti-viral, anti-cancer, anti-angiogenesis, anti-fungal, hemolytic, cytostatic, cholesterol-lowering, hypoglycemia and anti-dementia activities [4,7,13,14,15,16,17,18,19,20,21,22,23,24].

Saponins are amphipathic compounds that generally possess a triterpene or steroid backbone or aglycone. Triterpenoid saponins have aglycones that consist of 30 carbons, whereas steroidal saponins possess aglycones with 27 carbons, which are rare in nature [4].

Triterpene saponins belong to one of the most numerous and diverse groups of natural occurring products, which are produced in relatively high abundance. They are reported primarily as typical metabolites of terrestrial plants [25]. A few marine species belonging to the phylum *Echinodermata* [26] namely holothuroids (sea cucumbers) [7,10,13,27,28,29,30,31,32,33] and asteroids, and sponges from the phylum *Porifera* [13,34,35] produce saponins.

The majority of sea cucumber saponins, generally known as Holothurins, are usually triterpene glycosides, belonging to the holostane type group rather than nonholostane [36,37], which is comprised of a lanostane-3β-ol type aglycone containing a γ-18 (20)-lactone in the d-ring of tetracyclic triterpene (3β,20*S*-dihydroxy-5α-lanostano-18,20-lactone) [25] sometimes containing shortened side chains, and a carbohydrate moiety consisting of up to six monosaccharide units covalently connected to C-3 of the aglycone [7,8,13,37,38,39,40,41,42].

The sugar moiety of the sea cucumber saponins consists mainly of d-xylose, d-quinovose, 3-*O*-methyl-d-glucose, 3-*O*-methyl-d-xylose and d-glucose and sometimes 3-*O*-methyl-d-quinovose, 3-*O*-methyl-d-glucuronic acid and 6-*O*-acetyl-d-glucose [40,41,43,44,45,46,47,48]. In the oligosaccharide chain, the first monosaccharide unit is always a xylose, whereas either 3-*O*-methylglucose or 3-*O*-methylxylose is always the terminal sugar.

Although some identical saponins have been given different names by independent research groups [6] as they could be isomeric compounds, our comprehensive literature review showed that more than 250 triterpene glycosides have been reported from various species of sea cucumbers [7,13,18,25,29,41,44,49,50]. They are classified into four main structural categories based on their aglycone moieties; three holostane type glycoside group saponins containing a (1) 3β-hydroxyholost-9 (11)-ene aglycone skeleton; (2) saponins with a 3β-hydroxyholost-7-ene skeleton and (3) saponins with an aglycone moiety different to the other two holostane type aglycones (other holostane type aglycones); and (4) a nonholostane aglycone [25,38,46,51,52].

One of the most noteworthy characteristics of many of the saponins from marine organisms is the sulfation of aglycones or sugar moieties [4]. In sea cucumber saponins, sulfation of the oligosaccharide chain in the Xyl, Glc and MeGlc residues has been reported [38,40,46,53,54]. Most of them are mono-sulfated glycosides with few occurrences of di- and tri-sulfated glycosides. Saponin diversity can be further enhanced by the position of double bonds and lateral groups in the aglycone.

Triterpene glycosides have been considered a defense mechanism, as they are deleterious for most organisms [6,7,8,9,10,12,55,56,57]. In contrast, a recent study has shown that these repellent chemicals are also kairomones that attract the symbionts and are used as chemical “signals” [58]. However, in the sea cucumber, it has been suggested that saponins may also have two regulatory roles during reproduction: (1) to prevent oocyte maturation and (2) to act as a mediator of gametogenesis [18,59].

The wide range of biological properties and various physiological functions of sea cucumber extracts with high chemical structural diversity and the abundance of their metabolites have spurred researchers to study the ability of sea cucumbers to be used as an effective alternative source for potential future drugs. However, the large number of very similar saponin glycosides structures has led to difficulties in purification, and the complete structure elucidation of these molecules (especially isomers), has made it difficult to conduct tests to determine structure-activity relationships, which can lead to the development of new compounds with commercial applications [16]. Therefore, in order to overcome this problem, we employed High Performance Centrifugal Partition Chromatography (HPCPC) to successfully purify saponins in this study. HPCPC is more efficient in purifying large amounts of a given sample and also lower solvent consumption with high yields compared to other conventional chromatography methods.

This project aims to identify and characterize the novel bioactive compounds from the viscera (all internal organs other than the body wall) of an Australian sea cucumber *Holothuria lessoni* Massin *et al.* 2009 (golden sandfish) with an emphasis on saponins. *H*. *lessoni* was selected because it is a newly-identified Holothurian species, which is abundant in Australian waters. While only a few studies have compared the saponin contents of the body wall with that of the cuvierian tubules in other species [50,60,61,62], to our knowledge, no study has investigated the contribution of saponins of the body wall or the viscera of *Holothuria lessoni*. Sea cucumbers expel their internal organs as a defense mechanism called evisceration, a reaction that includes release of the respiratory tree, intestine, cuvierian tubules and gonads through the anal opening [50,58,61,63,64,65,66,67,68]. We hypothesize that the reason for this ingenious form of defense is because these organs contain high levels of compounds that repel predators [60,61,69,70]. Furthermore, the results of this project may identify the potential economic benefits of transforming viscera of the sea cucumber into high value co-products important to human health and industry. 

Matrix-assisted laser desorption/ionization time-of-flight mass spectrometry (MALDI-ToF/MS) and electrospray ionization mass spectrometry (ESI-MS) techniques allow the “soft” ionization of large biomolecules, which has been a big challenge until recently [71]. Therefore, MALDI and ESI-MS, and MS/MS were performed to detect saponins and to elucidate their structures.

## 2. Results and Discussion

An effective method for the purification of saponins has been developed, and several saponins were isolated and purified from the viscera of *H*. *lessoni*. The enriched saponin mixtures of the viscera extract were successfully purified further by HPCPC, which is very efficient in purifying compounds with low polarity as well as in processing large amounts of sample. This method yielded saponins with higher than a 98% recovery of sample with high purities [72]. Purifying saponins from mixtures of saponins also helps to overcome the problem associated with identifying multiple saponins with liquid chromatography-tandem mass spectrometry (LC-MS) and ESI-MS. Mass spectrometry has been applied for the structure elucidation of saponins in both negative and positive ion modes [73,74,75,76,77,78,79]. In this study, identification of the saponin compounds was attempted by soft ionization MS techniques including MALDI and ESI in the positive mode. Previous studies have reported that the fragment ions of alkali metal adducts of saponins provide valuable structural information about the feature of the aglycone and the sequence and linkage site of the sugar residues [80]. Therefore, the MS analyses were conducted by introducing sodium ions to the samples. However, saponin spectra can also be detected without adding a sodium salt. Because of the high affinity of alkali cations for triterpene glycosides, all saponins detected in the positive ion mode spectra were predominantly singly charged sodium adducts of the molecules [M + Na]^+^ [19,81]. The main fragmentation of saponins generated by cleavage of the glycosidic bond yielded oligosaccharide and monosaccharide fragments [19]. Other visible peaks and fragments were generated by the loss of other neutral moieties such as CO_2_, H_2_O or CO_2_ coupled with H_2_O.

The saponins obtained from the viscera of this tropical holothurian were profiled using MALDI-MS and ESI-MS. MALDI is referred to as a “soft” ionization technique, because the spectrum shows mostly intact, singly charged ions for the analyte molecules. However, in some cases, MALDI causes minimal fragmentation of analytes [71].

**Figure 1:**
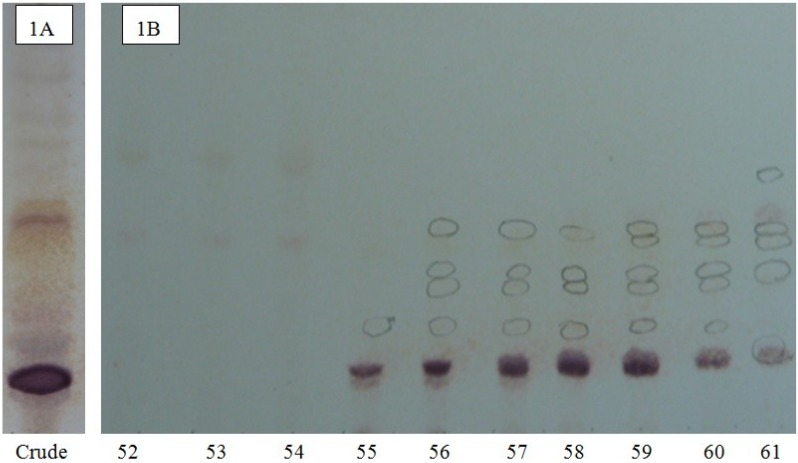
The thin-layer chromatography (TLC) pattern of a saponin mixture (**A**) and the high performance centrifugal partition chromatography (HCPCP) fractions (**B**) from the purified extracts of the viscera of the *Holothuria lessoni* sea cucumber using the lower phase of CHCl_3_-MeOH-H_2_O (7:13:8) system. The numbers under each lane indicate the fraction number of fractions in the fraction collector. Here, only the fractions 52 to 61 of one analysis (of 110 fractions) are shown as a representative.

The chromatographic purification of isobutanol-soluble saponin-enriched fractions of *H*. *lessoni* viscera was monitored on pre-coated thin-layer chromatography (TLC) plates (Figure 1A) showing the presence of several bands. As a typical example, the TLC profile of HPCPC Fractions 52–61 of the isobutanol-saponin enriched fraction from the viscera of the *H*. *lessoni* sea cucumber is shown in Figure 1B. The centrifugal partition chromatography (CPC) technique not only allowed for the purification of saponins, but in some cases it could separate isomeric saponins e.g., separation of the isomers detected in the ion peak at *m/z* 1303.6, which will be discussed later.

**Figure 2:**
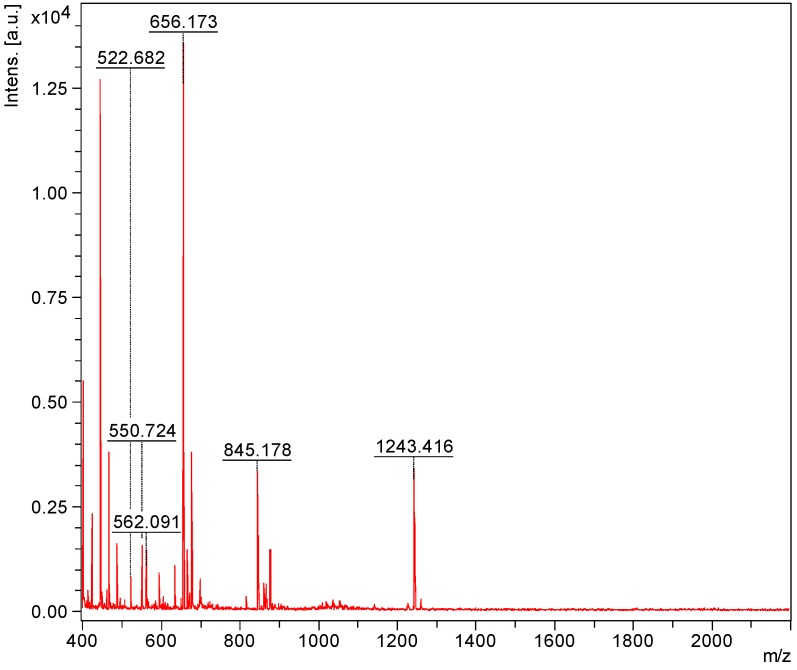
The full-scan matrix-assisted laser desorption/ionization mass spectrometry (MALDI) mass spectrum of HPCPC Fraction 55 in the (+) ion mode.

Mass spectrometry has been used extensively for the characterization of saponins and their structural confirmation. One of the powerful methods, which are widely used for the analysis of high molecular weight, non-volatile molecules is MALDI [82]. The appropriate HPCPC fractions were consequently pooled based on their TLC profiles and concentrated to dryness and analyzed by MALDI MS and MS/MS, and ESI MS/MS. In the positive ion mode, all detected ions were sodium-coordinated species such as [M + Na]^+^ corresponding to sulfated and non-sulfated saponins [64]. The prominence of the parent ions [M + Na]^+^ in MS spectra also enables the analysis of saponins in mixtures or fractions. The MALDI results indicate that the saponin fractions are quite pure, which is consistent with the TLC data. As a representative example, the full-scan MALDI mass spectrum of the saponin extract obtained from HPCPC Fraction 55 of the *H*. *lessoni* viscera is shown in Figure 2.

**Figure 3:**
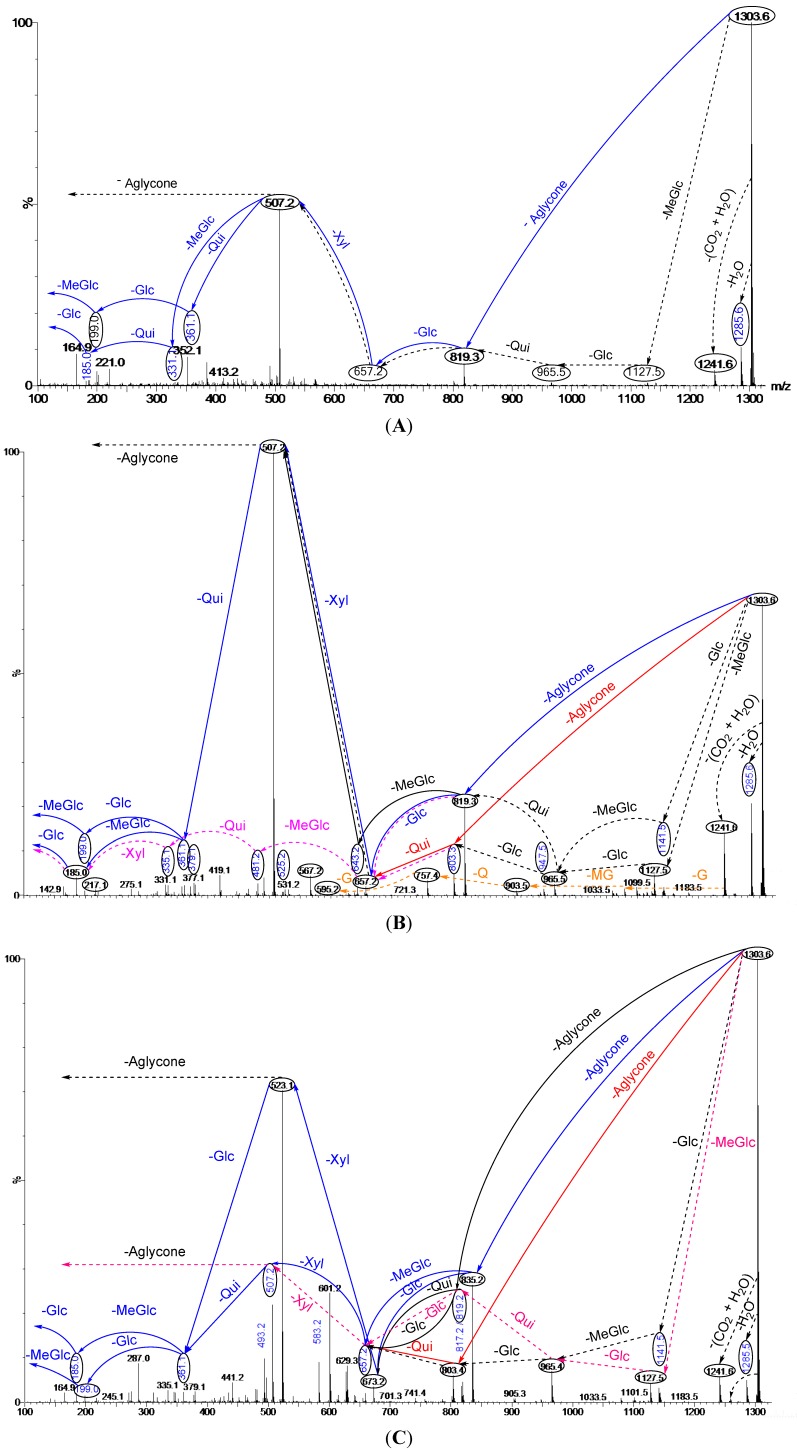
Schematic fragmentation patterns of the ion detected at *m/z* 1303.6; (**A**) Fraction 15; (**B**) Fraction 14 and (**C**) Fraction 12. Full and dotted arrows show the two main feasible fragmentation pathways. The predominant peak (**A** and **B**) at *m/z* 507 corresponds to the key sugar residue and aglycone moiety. The major abundant peak (**C**) at *m/z* 523 corresponds to both the key sugar residue and aglycone moiety. Abbreviations; G = Glc, MG = MeGlc, Q = Qui, X = Xyl.

This spectrum displays the major intense peak detected at *m/z* 1243.4, which corresponds to Holothurin A, with an elemental composition of C_54_H_85_NaO_27_S [M + Na]^+^. Other visible peaks seem to correspond to the sugar moieties and aglycone ions generated by the losses of sugars and/or losses of water and/or carbon dioxide from cationized saponins upon MALDI ionization. These analyses show that this fraction contains one main saponin. Therefore, even though the HPCPC fractionation separated the saponin mixture, some saponin congeners, due to the similarity in their TLC migration, were detected in some of the pooled fractions. It was found that the total separation of the saponins was difficult within a single HPCPC run. However, this technique allowed the separation of a number of saponins, including some isomers (Figure 3).

**Figure 4:**
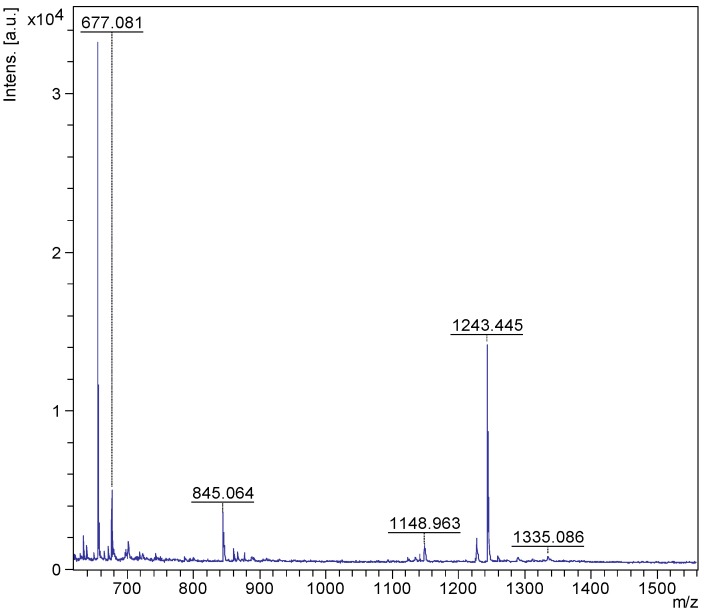
The full-scan MALDI mass spectrum of the isobutanol-enriched saponin extract from the viscera of the *H*. *lessoni*. A mass range of 600 to 1500 Da is shown here. It is noted that this spectrum is unique for this species.

**Figure 5:**
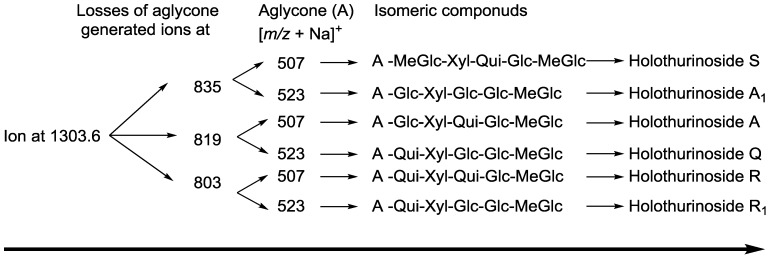
The schematic diagram of the proposed isomeric structures of ion at *m/z* 1303.6.

**Figure 6:**
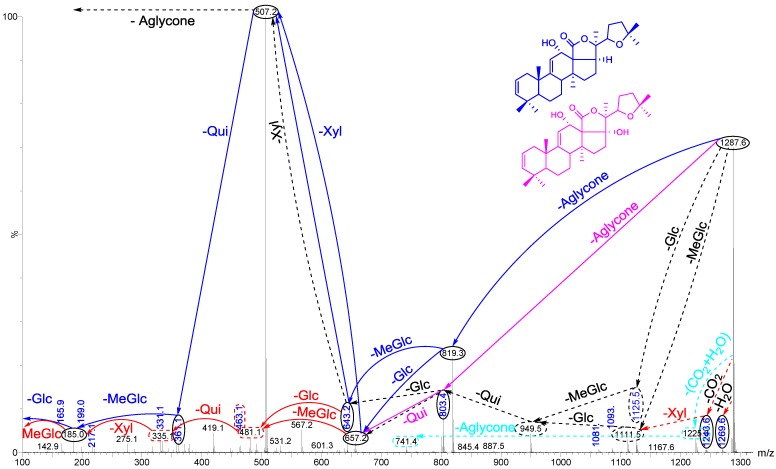
(+) ion mode ESI-MS/MS spectrum of saponins detected at *m/z* 1287.6. This spectrum shows the presence of two different aglycones, which led to the isomeric saponins. Full and dotted arrows illustrate the two main possible fragmentation pathways.

**Figure 7:**
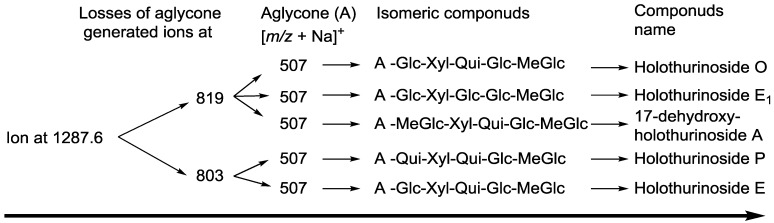
A schematic diagram of the proposed isomeric structures of ion at *m/z* 1287.6.

The full-scan MALDI mass spectrum of the isobutanol-enriched saponin extract obtained from the viscera of the *H*. *lessoni* is shown in Figure 4. A diverse range of saponins with various intensities was identified. This spectrum displays 13 intense peaks that could each correspond to at least one saponin congener. The most abundant ions observed under positive ion conditions were detected at *m/z* 1335, 1303, 1289, 1287, 1259, 1245, 1243, 1229, 1227, 1149, 1141, 1123 and 845. Further analysis revealed that some of these MS peaks represented more than one compound. For instance the peaks at *m/z* 1303 and 1287 were shown to contain at least six and five different congeners, respectively (Figure 3 and Figure 5, Figure 6, Figure 7).

The accurate mass measurements acquired by MALDI-MS detected the saponin peaks, and molecular formulae and elemental compositions were assigned by ESI-MS/MS as summarized in Table 1. Our results revealed that at least 75 saponins were detected in *H*. *lessoni*, including 39 new sulfated, non-sulfated and acetylated triterpene glycosides, containing a wide range of aglycone and sugar moieties.

**Table 1:** Summary of saponins identified from the viscera of *H*. *lessoni* by matrix-assisted laser desorption/ionization time-of-flight mass spectrometry (MALDI-ToF-MS) and electrospray-ionization mass spectrometry (ESI-MS). This table illustrates the 39 novel identified compounds (N) along with the 36 known compounds (P). This table also shows some identical saponins, which have been given different names by different researchers in which they might be isomeric congeners.

[M + Na]^+^ *m/z*	MW	Formula	Compound’s Name	Novel (N)/Published (P)	References
889.4	866	C_41_H_63_NaO_16_S	Holothurin B_3_	P	[83]
		C_42_H_67_NaO_15_S	Unidentified	N	−
905.4	882	C_41_H_63_NaO_17_S	Holothurin B_4_	P	[83]
			Holothurin B	P	[61,84,85,86]
			Nobiliside B	P	[87]
907.4	884	C_41_H_65_NaO_17_S	Holothurin B_2_	P	[83]
			Leucospilotaside B	P	[8]
911.6	888	C_45_H_92_O_16_	Unidentified	N	−
917.4	994	C_44_H_71_NaO_15_S	Unidentified	N	−
921.4	898	C_41_H_63_NaO_18_S	Leucospilotaside A	P	[84]
1034.1	1011	a*	Unidentified	N	−
1065.5	1042	C_48_H_82_O_24_	Unidentified	N	−
1071.5	1048	C_47_H_93_NaO_21_S	Unidentified	N	−
1078.5	1055	a *	Unidentified	N	−
1083.3	1060	C_58_H_64_O_25_	Unidentified	N	−
1087.6	1064	C_47_H_93_NaO_22_S	Unidentified	N	−
1123.5	1100	C_54_H_84_O_23_	Unidentified	N	−
1125.5	1102	C_54_H_86_O_23_	Holothurinoside C Holothurinoside C_1_	P	[62,69,88,89]
1127.6	1104	C_54_H_88_O_23_	Unidentified	N	−
			Unidentified	N	−
1141.6	1118	C_54_H_86_O_24_	Desholothurin A (Nobiliside 2a), Desholothurin A_1_ (Arguside E)	P	>[5,62,69,88,89,90]
1149.2	1126	a *	Unidentified	N	−
1157.5	1134	C_54_H_109_O_25_	Holothurinoside J_1_	P	[50]
		C_49_H_91_NaO_25_S	Unidentified	N	−
1193.5	1170	C_55_H_87_NaO_23_S	Unidentified	N	−
1199.4	1176	C_54_H_64_O_29_	Unidentified	N	−
1221.5 **	1198	C_56_H_78_O_28_	Unidentified	N	−
1225.5	1202	C_54_H_83_NaO_26_S	Unidentified	N	−
1227.5	1204	C_54_H_85_NaO_26_S	Fuscocineroside B/C, Scabraside A or 24-Dehydroechinoside A	P	[29,56,89,91,92]
1229.5	1206	C_54_H_87_NaO_26_S	Holothurin A_2_, Echinoside A	P	[7,61,91,93,94,95]
1243.5	1220	C_54_H_85_NaO_27_S	Holothurin A Scabraside B 17-Hydroxy fuscocineroside B 25-Hydroxy fuscocinerosiden B	P	[29,58,61,95,96,97]
1245.5	1222	C_54_H_87_NaO_27_S	Holothurin A_1 _	P	[91]
			Holothurin A_4 _		[36]
			Scabraside D		[92]
1259.5	1236	C_54_H_85_NaO_28_S	Holothurin A_3_	P	[36]
			Unidentified	N	−
1265.5	1242	C_56_H_83_NaO_27_S	Unidentified	N	−
1271.6	1248	C_60_H_96_O_27_	Impatienside B	P	[5,98]
1287.6	1264	C_60_H_96_O_28_	Holothurinoside E, Holothurinoside E_1_	P	[62,69]
			Unidentified	N	−
			Unidentified	N	−
			17-Dehydroxyholothurinoside A	P	[5,99]
1289.6	1266	C_60_H_98_O_28_	Griseaside A	P	[99]
1301.6	1278	C_61_H_98_O_28_	Holothurinoside M	P	[65]
		C_60_H_94_O_29_	Unidentified	N	−
1303.6	1280	C_60_H_96_O_29_	Holothurinoside A	P	[5,62,69,88]
			Holothurinoside A_1_		
			Unidentified	N	−
			Unidentified	N	−
			Unidentified	N	−
			Unidentified	N	−
1305.6	1282	a *	Unidentified	N	−
1317.6	1294	C_61_H_98_O_29_	Unidentified	N	−
1335.3	1312	a *	Unidentified	N	−
1356.4	1333	a *	Unidentified	N	−
1409.4	1386	C_61_H_78_O_36_	Unidentified	N	−
1411.7	1388	C_62_H_116_O_33_	Unidentified	N	−
1419.7	1396	C_66_H_108_O_31_	Unidentified	N	−
1435.7	1412	C_66_H_108_O_32_	Unidentified	N	−
1465.7	1442	C_67_H_110_O_33_	Arguside B	P	[5,32]
			Arguside C		
1475.6	1452	C_65_H_96_O_36_	Unidentified	N	−
1477.7 **	1454	C_61_H_114_O_38_	Unidentified	N	−
1481.7	1458	C_66_H_106_O_35_	Unidentified	N	−
1493.7	1470	C_65_H_114_O_36_	Unidentified	N	−
1495.7	1472	C_61_H_116_O_39_	Holothurinoside K_1_	P	[50]
		C_72_H_112_O_31_	Unidentified	N	−
1591.7	1568	C_66_H_120_O_41_	Unidentified	N	−

a * The composition was not measured through the ESI analysis; ** acetylated compounds.

A number of studies have reported the presence of multiple saponins. Elbandy *et al.* [5] described the structures of 21 non-sulfated saponins from the body wall of *Bohadschia cousteaui*. These authors reported 10 new compounds together with 11 known triterpene glycosides including Holothurinoside I, Holothurinoside H, Holothurinoside A, Desholothurin A, 17-dehydroxyholothurinoside A, Arguside C, Arguside F, Impatienside B, Impatienside A, Marmoratoside A and Bivittoside. Bondoc *et al.* [64] investigated saponin congeners in three species from Holothuriidae (*H*. *scabra* Jaeger 1833, *H*. *fuscocinerea* Jaeger 1833, and *H*. *impatiens* Forskal 1775). This group reported 20 saponin ion peaks, with an even number of sulfated and non-sulfated types, in *H*. *scabra,* which contained the highest saponin diversity among the examined species, followed by *H*. *fuscocinerea* and *H*. *impatiens* with 17 and 16 saponin peaks, respectively. These authors also described a total of 32 compounds in *H*. *scabra* and *H*. *impatiens* and 33 compounds in *H*. *fuscocinerea*. The saponin content of five tropical sea cucumbers including *H*. *atra*, *H*. *leucospilota*, *P*. *graeffei*, *A*. *echinites* and *B*. *subrubra* was also studied by Van Dyck *et al.* [50]. These authors reported the presence of four, six, eight, ten and nineteen saponin congeners in these species, respectively. In addition, this group [69] also detected a higher number of saponins (26) in the cuvierian tubules of *H*. *forskali* compared to the body wall (12 saponins). These results further support the evidence, suggested by the present study, of greater saponin congeners in viscera.

### 2.1. MALDI-MS/MS Data of Compound Holothurin A in the Positive Ion Mode

**Figure 8:**
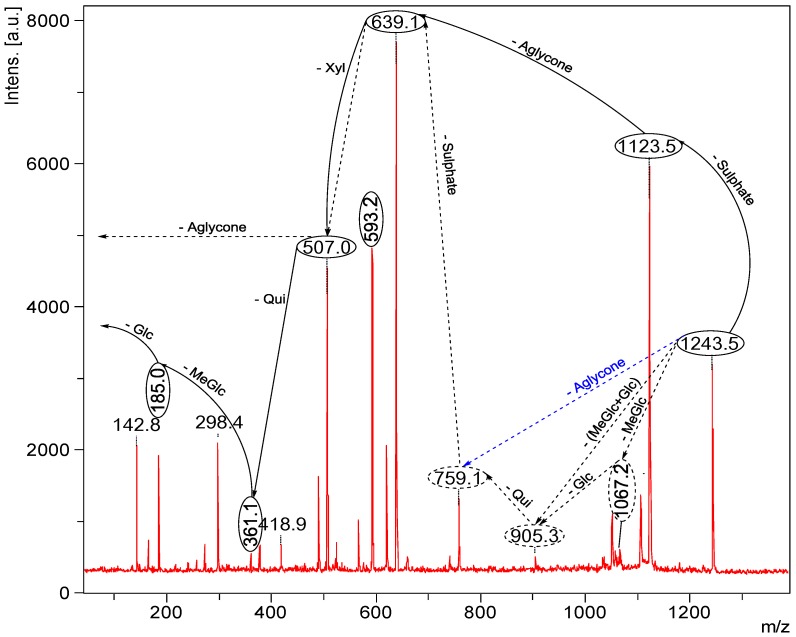
Positive tandem MALDI spectrum analysis of the precursor ion (saponin) detected at *m/z* 1243.5. The figure shows the collision-induced fragmentation of parent ions at *m/z* 1243.5. The consecutive losses of sulfate group, aglycone, xylose (Xyl), quinovose (Qui) and 3-*O*-methylglucose (MeGlc) residues affords product ions detected at *m/z* 1123, 639, 507, 361and 185, respectively.

The conventional procedures to differentiate between isomeric saponins, including chemical derivatization and stereoscopic analysis, are tedious and time-consuming [100]. Tandem mass spectrometry was conducted to obtain more structural information about the saccharide moiety and elucidate their structural features. In order to ascertain that ions (signals) detected in the full-scan MALDI MS spectrum indeed correspond to saponin ions, tandem mass spectrometry analyses were performed for each ion, and saponin ion peaks were further analyzed using MS/MS fingerprints generated with the aid of collision-induced dissociation (CID) from their respective glycan structures. CID can provide a wealth of structural information about the nature of the carbohydrate components, as it preferentially cleaves glycosides at glycosidic linkages, allowing a straightforward interpretation of data. Almost all of observed daughter ions originated from the cleavage of glycosidic bonds (Figure 8). Therefore, the reconstruction of their fingerprints (fragmentation patterns) created by the glycosidic bond cleavages was utilized to deduce the structure of sugar moieties. This technique was also able to distinguish the structural differences between the isomers following HPCPC separation. However, in some cases, the MS/MS spectra obtained from the CID could be essentially identical for isomeric precursor ions. As a typical example, the MALDI-MS/MS mass spectrum for the ion detected at *m/z* 1243.5 is shown in Figure 8. The fragmentation pattern of the sodiated compound at *m/z* 1243.5 [M + Na]^+^ in successive MS experiments is discussed in detail below for stepwise elucidation of the molecular structure of these compounds. 

Collisional induced-dissociation activates two feasible fragmentation pathways of cationized parent ions shown in full and dotted arrows. First, the loss of the sugar unit; the successive losses of 3-*O*-methylglucose (-MeGlc), glucose (-Glc), quinovose (-Qui), sulfate and xylose (-Xyl) units generate ion products detected at *m/z* 1067, 905, 759, 639 and 507, respectively. As this figure illustrates, the consecutive losses of the (MeGlc + Glc) simultaneously generated the ion at *m/z* 905.3, and Qui (−146 Da) resulted in the peak at *m/z* 759.1 which corresponds to [Aglycone + sulXyl-H + 2Na]^+^.

Secondly the decomposition of the precursor ions can also be triggered by the loss of the aglycone residue, creating peaks at *m/z* 759 (Figure 8) corresponding to the sugar moieties of 1243.5. The losses of the NaHSO_4_ (ion generated at *m/z* 1123.5), aglycone residue (ion generated at *m/z* 639.1), and xylose (ion generated at *m/z* 507.0), respectively, were produced by glycone and aglycone fingerprint peaks from the precursor ion. Therefore, the consecutive losses of the sodium monohydrogen sulfate (NaHSO_4_) from 1243.5 and aglycone unit produced signals observed at *m/z* 1123 and 639 (Figure 8); the latter peak corresponding to the total desulfated sugar moiety. Furthermore, the consecutive losses of Xyl, Qui and MeGlc presenting signals observed at *m/z* 507, 361 and 185, respectively, additionally proved that the decomposing ions were definitely generated from sodiated Holothurin A (*m/z* 1243.5). The implementations of these molecular techniques on all ions detected in the MALDI spectra allow us to identify the molecular structures of the saponins. All spectra were analyzed and fragmented, and some of them shared common fragmentation patterns. Key fragments from the tandem MS spectra of the positive ion mode of MALDI and ESI were reconstructed according to the example illustrated in order to propose the saponin structures. On the bases of these fragment signatures, 39 new saponins can be postulated. Some of these compounds, which share the common *m/z* 507 or/and *m/z* 523 key signals as a signature of the sodiated MeGlc-Glc-Qui and the sodiated MeGlc-Glc-Glc oligosaccharide residues, respectively, were easily identified. The identified saponins possess different aglycone structural elements.

The loss of 18 Da from the sodiated molecular ion, suggested the elimination of a neutral molecule (H_2_O) from the sugar group [19]. The simultaneous loss of two sugar units indicated characteristics of a branched sugar chain. Other visible peaks correspond to saponin product ions produced by the losses of water and/or carbon dioxide from sodiated saponins upon MALDI ionization. Hereby the sugar sequence of saponins can be determined by applying CID. The MALDI MS/MS data for this *m/z* value were in complete agreement with those reported in a previous study [50,64]. The predominant fragment signal at *m/z* 593.2 results from α ^1,5^A_4_ cross-ring cleavage of the sulXyl residue, which was consistent with previous findings for the MS/MS analyses of sea cucumber saponins [64]. However, this peak was only detected as an intense signal in the sulfated saponins such as Holothurin A, whereas it was not observed in the non-sulfated saponins such as Holothurinoside A. Therefore, this cross-ring cleavage seems to occur only with the sulfated Xyl. Analysis by MALDI resulted in an information rich tandem mass spectrum containing glycosidic bond and cross-ring cleavages that provided more structural information than previous studies on the same precursor ion. The sugar moiety of saponins developed from non-sulfated hexaosides to sulfated tetraosides [64]. The assignment of the sulfate group was determined by the mass difference between the parent ion at *m/z* 1243 and daughter ion *m/z* 1123 peaks based on knowing the molecular weight of the sulfate unit (120 Da). Complete glycosidic bond cleavage was observed, which enabled us to determine the locations of the sulfate (*m/z* 1123), the entire sugar moieties (*m/z* 639), and each component of sugar residue.

The losses of the aglycone and sugar residues are largely observed from glycosidic bond cleavages. Even though one cross-ring cleavage is assigned, the generation of glycosidic bond cleavages in combination with accurate mass is sufficient to assign the position of the sulfate group along the tetrasaccharide sequence for Holothurin A. The ion detected at *m/z* 1105 (Figure 8) is the water-loss ion derived from the ion at *m/z* 1123, whereas the ion observed at *m/z* 1061 corresponds to the neutral loss of CO_2_ (44 Da).

As described by Song *et al.* [100], the cross-ring cleavages that occurred in the CID spectra of saccharides with α 1–2 linkage, such as the sugar residue for Holothurin A, are X and A types, whereas the glycoside bond cleavages are C and B types. The major peak at *m/z* 593.2 was attributed to cross-ring cleavage of the sugar unit. 

This MS/MS spectrum allows us to reconstruct the collision-induced fragmentation pattern of the parent ion (Figure 4) and consequently to confirm that ions monitored at *m/z* 1243.5 correspond to the Holothurin A elucidated by Van Dyck *et al.* [50], Kitagawa *et al.* [96] and Rodriguez *et al.* [88].

The occurrence of a sulfate group (NaHSO_4_) in saponin compounds, such as in the case of Holothurin A, was assigned by a loss of 120 Da during the MS/MS. By the combination of accurate mass and MS/MS information, saponins were categorized into seven distinct carbohydrate structural types: (A) MeGlc-Glc-Qui-Xyl-Aglycone; (B) MeGlc-Glc-Glc-Xyl-Aglycone; (C) (MeGlc-Glc)-Qui-sulXyl-Aglycone; (D) MeGlc-Glc-Qui-(Qui-Glc)-Xyl-Aglycone; (E) MeGlc-Glc-Qui-(MeGlc-Glc)-Xyl-Aglycone; (F) MeGlc-Glc-Glc- (MeGlc-Glc)-Xyl-Aglycone; and (G) MeGlc-Glc-Glc-(Qui-Glc)-Xyl-Aglycone. Non-sulfated saponins had one to six monosaccharide units and six distinct structural types. All sulfated saponins ranging from *m/z* 889 to 1259 had a structure (C), in which Xyl was sulfated. However, in some cases, the sulfation of Xyl, MeGlc and Glc was reported [13]. The MS analyses also indicated that this sea cucumber species produced a mixture of common and unique saponin types. Unique saponin types were also identified when the mass spectra of this species were compared with others. Saponin peaks with the ion signatures at *m/z* values of 1477, 1335, 1221, 1149 and 1123 were unique in *H*. *lessoni*. In the tandem MS, in general, the most abundant ions were attributed to the losses of aglycones and/or both key diagnostic sugar moieties (507 and 523). For 1243.5, the most abundant ions observed under positive ion conditions were at *m/z* 1123, 639 and 507, corresponding to the losses of sulfate, aglycone and Xyl moieties. The major ion at *m/z* 621.2 corresponded to the loss of water from ion at *m/z* 639. Some saponins were commonly found among species (e.g., Holothurins A and B), whereas others were unique to each species (e.g., 1221 in *H*. *lessoni*), as Bondoc *et al.* [64] and Caulier *et al.* [6] have also indicated. The saponin profile (peaks) of sea cucumbers indicated the different relative intensities of saponins in the viscera. The peaks observed (Figure 4) at *m/z* 1149.0, 1227.5, 1229.5, 1243.5, and 1259.5 in the positive ion mode corresponded to an unidentified saponin, Scabraside A or Fuscocinerosides B/C (isomers), Holothurin A_2_ (Echinoside A), Holothurin A, and Holothurin A_3,_ respectively [36,56,61,89,93,94]. Most of these sulfated saponins were also reported by Kitagawa *et al.* [89] and Bondoc *et al.* [64]. The ion peaks of the non-sulfated saponins at *m/z* 1125, 1141, 1287, 1289, 1301 and 1303 corresponded to Holothurinosides C/C_1_ (isomers), Desholothurin A (synonymous with Nobiliside 2A) or Desholothurin A_1_, Holothurinosides E/E_1_, Griseaside A, Holothurinosides M and A, respectively [69]. *H*. *scabra*, *H*. *impatiens* and *H*. *fuscocinerea* were also reported to contain Holothurin A, Scabraside B and Holothurinoside C [64]. This group also detected 24-dehydroechinoside A and Scabraside A in *H*. *scabra*. The presence of Holothurinosides C/C_1_ (isomers), Holothurinosides A/A_1_ (isomers), Desholothurin A (synonymous with Nobiliside 2A), Desholothurin A_1_ and Holothurinosides E/E1 were also described in *H*. *forskali* by several groups [65,69,88]. We were not able to identify all the saponin congeners detected in the semi-pure extract in the HPCPC-fractionated samples. Bondoc *et al.* [64] experienced a similar issue in that they observed some peaks in MALDI MS, which were not seen in the isomeric separation done in LC-ESI MS. For instance, we could not find ions at *m/z* 1149 and 1335 in the spectra of HPCPC fractions by ESI-MS. The MALDI mass spectra of the semi-pure and HPCPC fractionated samples of the *H*. *lessoni* revealed 75 ions (29 sulfated and 46 non-sulfated) in which a total of 13 isomers was found (Table 1), of which 36 congeners had previously been identified in other holothurians. It is the first time that the presence of these identified saponins has been reported in *H*. *lessoni,* apart from the saponins reported by Caulier *et al.* [58] that were found in the seawater surrounding *H*. *lessoni*. They reported saponins with *m/z* values of 1141, 1229, 1243 and 1463 namely Desholothurin A, Holothurin A_2_, Scabraside B (synonymous with Holothurin A) and Holothurinoside H, respectively [58]. However, we could not detect the ion at *m/z* 1463 in our sample.

Most of the sulfated saponins that had previously been reported were detected in this species, including Holothurin B_3_ (*m/z* 889), Holothurin B/B_4_ (*m/z* 905), Holothurin B_2_ (*m/z* 907), Fuscocinerosides B or C, which are functional group isomers (*m/z* 1227), Holothurin A_2_ (*m/z* 1229), Holothurin A (*m/z* 1243), Holothurin A_1_/A_4_ (*m/z* 1245), and Holothurin A_3_ (*m/z* 1259). The common sulfated congeners among this species and other sea cucumbers are Holothurin B (*m/z* 905) and Holothurin A (*m/z* 1243). Among these saponins, Holothurin A is the reported to be the major congener with the highest relative abundance in this species.

To illustrate the identification of a novel compound at *m/z* 1149.0, the parent ion at *m/z* 1149.0 was subjected to MS/MS fragmentation. The MALDI fingerprints revealed that the compound contained a novel aglycone at *m/z* 493 and a tetrasaccharide moiety with *m/z* value of 656 Da including -Xyl, -Qui, -Glc and -MeGlc in the ratio of 1:1:1:1. This saponin possessed the common *m/z* 507 key signal as a fingerprint of MeGlc-Glc-Qui + Na^+^. We propose to name Holothurinoside T.

The isomers within one sample showed different MS^n^ spectra [101] allowing their structures to be elucidated based on the ion fingerprints. Here we indicate that the occurrence of many product ions in the spectrum of viscera extract is due to the presence of a mixture of saponins and isomeric saponins (Figure 3 and Figure 5, Figure 6, Figure 7). This observation is consistent with the findings proposed by Van Dyck and associates [69] for the Cuvierian tubules of *H*. *forskali*. Mass spectrometry alone, however, is not powerful enough to obtain more structural information about the isomeric congeners. Nonetheless, it provides a quick and straightforward characterization of the element components and saponin distributions by the presence of ions at *m/z* 507 and 523 in the tandem spectra of the viscera extracts. 

### 2.2. Key Fragments and Structure Elucidation of Novel Saponins

The common key fragments facilitated the structure elucidation of novel saponins. Tandem mass spectrometry analyses of saponins led to identification of several diagnostic key fragments corresponding to certain common structural element of saponins as summarized in Table 2.

**Table 2:** Key diagnostic ions in the MS/MS of the holothurians saponins.

Diagnostic ions in CID Spectra of [M + Na]^+^			
*m/z* Signals (Da)			
	507	523	639
Chemical signatures	MeGlc-Glc-Qui + Na	MeGlc-Glc-Glc + Na	MeGlc-Glc-Qui-Xyl + Na

The structures of saponins were deduced by the identification and implementation of the key fragment ions generated by tandem mass spectrometry. The presence of these oligosaccharide residues (*m/z* 507 and/or 523) facilitated the determination of the saponin structure. However, some compounds with a *m/z* *v*alue of less than 1100 Da including 921, 907, 905 and 889 did not yield the peak *m/z* 523, which reflected the lack of this oligosaccharide unit in their structures. Unlike other compounds, the MS/MS spectrum of the ion at *m/z* 1477.7 illustrated the unique fingerprint profile, which contained ions at *m/z* 511 and 493 instead of an ion at m/z 507. The structure of compound was further confirmed by MS/MS analyses.

The MALDI analysis revealed that the ion with *m/z* 1243.5 was the prominent peak in the spectrum, which corresponded to Holothurin A, which was found in several species of sea cucumbers [6,29,50,58,61,64,89,95,96,97]. The MALDI data were confirmed by ESI-MS.

**Figure 9:**
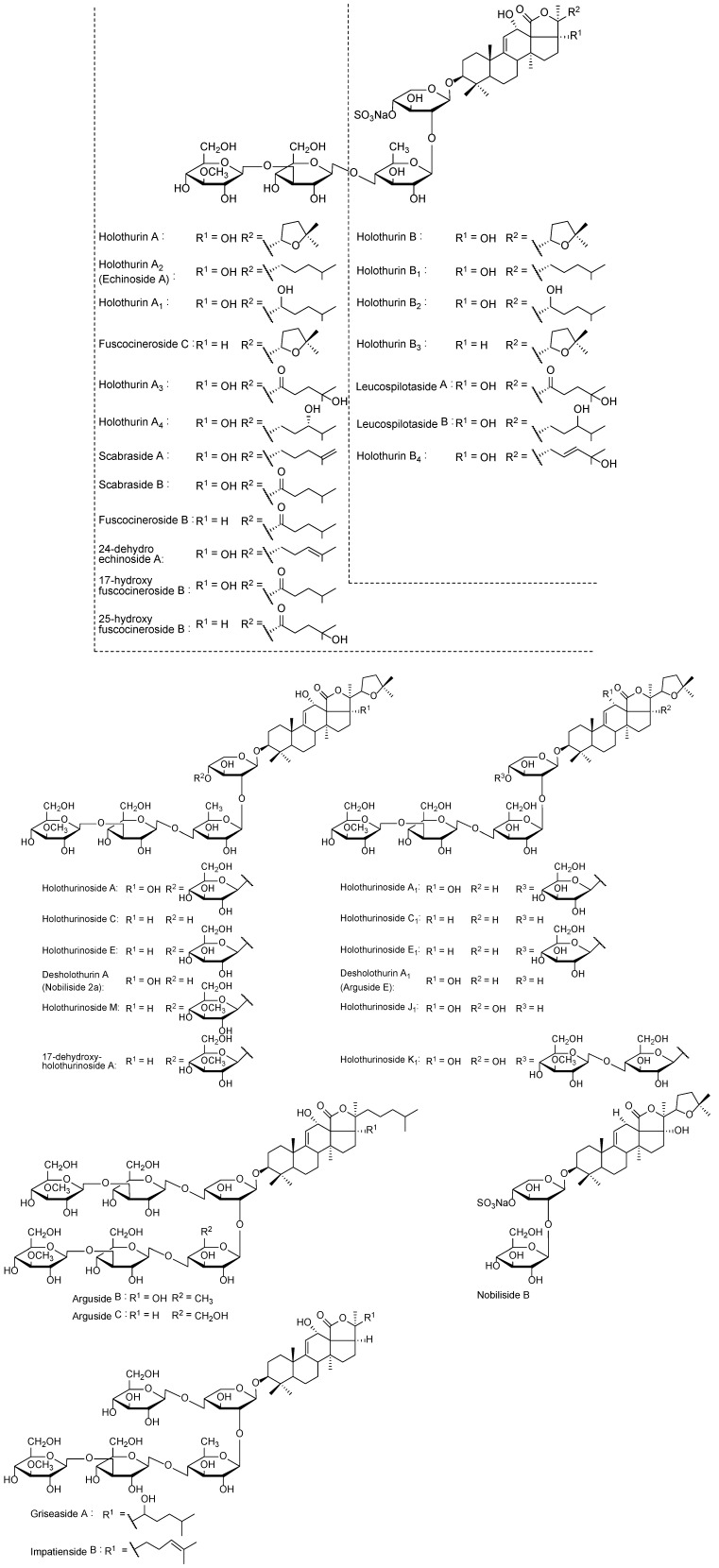
The structure of identified saponins in the viscera of *H*. *lessoni*.

Table 1 summarizes data of all analyses performed on the saponin-enriched sample and HPCPC fractionated samples using MALDI and ESI on compounds from the viscera of *H*. *lessoni*. The identified saponin mixture contains a diverse range of molecular weights and structures. The chemical structures of the identified compounds are illustrated in Figure 9. The isobutanol and HPCPC fractionated samples indicated 29 sulfated and 46 non-sulfated saponin ions. The number of MS ion peaks was lower than the number of isomers identified by MS/MS following HPCPC separation (Figure 3). 

### 2.3. Analyses of Saponins by ESI-MS

The positive ion mode ESI-MS analyses were also conducted on the samples. ESI mass spectra of the saponins are dominated by [M + Na]^+^. There were some instances where peaks observed in the MALDI-MS spectra were not monitored from the isomer separation done in the ESI-MS, such as the peak detected at *m/z* 1149 in the MALDI spectra. Other researchers had experienced the same issue [50,64]. 

**Figure 10:**
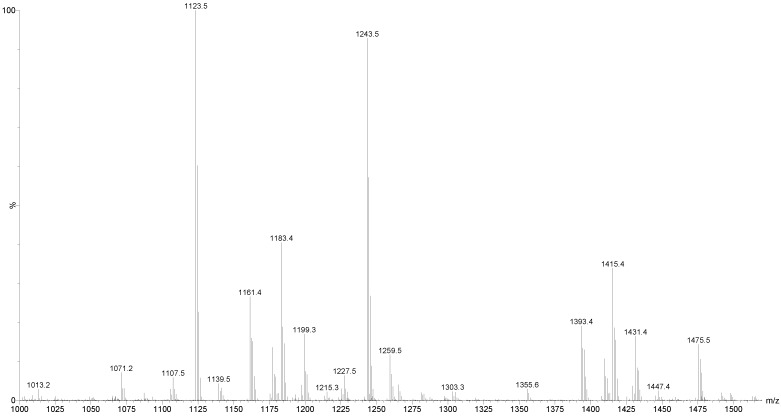
(+) ESI-MS spectrum of saponins extract from the viscera of *H*. *lessoni*.

ESI-MS^n^ is a very effective and powerful technique to differentiate isomeric saponins [100]. Tandem MS analyses on [M + Na]^+^ ions provided abundant structural information about saponins. The positive ion mode ESI-MS/MS analyses were also performed on all compound ions detected in the ESI-MS spectrum of HPCPC fractions. This technique also confirmed the existence of saponins reported in the literature and allowed the discovery of new saponin congeners in the species examined. The molecular masses of the identified compounds are summarized in Table 1. The ESI-MS spectrum of the saponin extract from the viscera of *H*. *lessoni* is shown in the Figure 10.

**Figure 11:**
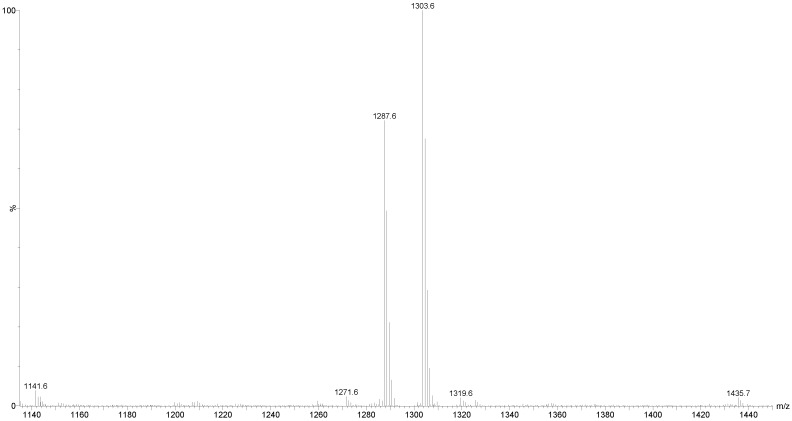
(+) ion mode ESI-MS spectrum of saponins extract from Fraction 14.

Several major peaks were detected. The peaks at *m/z* 1123 and 1243 correspond to a novel compound and Holothurin A with the elemental compositions of C_54_H_84_O_23_ and C_54_H_85_NaO_27_S, respectively. The ESI-MS analyses were also carried out on all HPCPC fractions. As a typical example, Figure 11 shows the ESI-MS spectrum of Fraction 14.

As can be seen in Figure 11, there are two major peaks at *m/z* 1287.6 and 1303.6, which correspond to Holothurinosides E/E_1_ and Holothurinosides A/A_1_, respectively. These two peaks, as the MS/MS analyses show which will be discussed later, were found to correspond to at least five and six isomers, respectively (Figure 3, Figure 6 and Supplementary Figure S2). A comparison of the molecular weights of both saponins revealed some mass differences between them, such as a 16 Da (O) mass differences between Holothurinosides E/E_1_ and Holothurinosides A/A_1_, reflecting the small structural alterations and the intrinsic connections between them. Their MS/MS analyses indicated, as will be discussed later, the presence of some identical aglycones in both ions. 

#### 2.3.1. Molecular Mass of Saponins by ESI

ESI/MS provide considerable structural information with very high sensitivity for saponins [60,63]. Peaks corresponding to the sodium adduct of the complete sugar side chains were often quite intense in the product ion spectra of the sodiated saponin precursor. Tandem mass spectra of saponins reflected the different fingerprints with different relative intensities.

#### 2.3.2. Structure Elucidation of the Saponins by ESI-MS/MS

**Figure 12:**
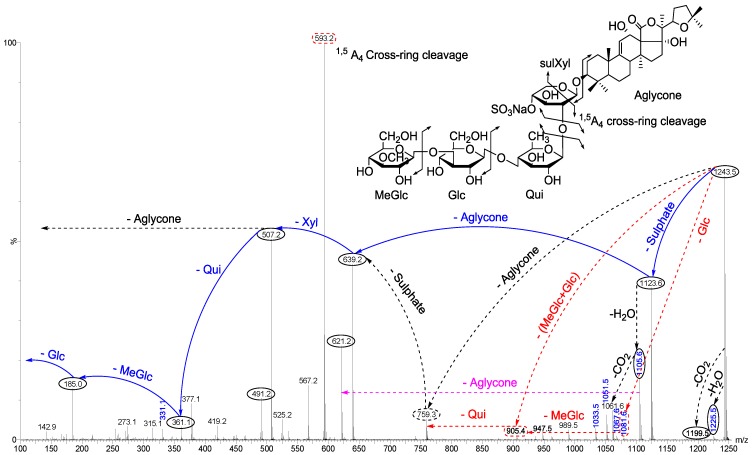
(+) Ion mode ESI-MS/MS spectrum of saponin detected at 1243.5 (Holothurin A). Full and dotted arrows show the two main feasible fragmentation pathways. The structure of saponin was elucidated on the base of tandem mass spectrometry.

Seventy-five different triterpene saponins purified from sea cucumber were investigated by MALDI and electrospray ionization tandem mass spectrometry (ESI-MS/MS) in the positive ion modes. All spectra were analyzed and fragmented, and some of them shared common fragmentation patterns. Key fragments from the positive ion mode MS/MS spectra of MALDI and ESI were reconstructed with an example illustrated that proposes the saponin structures. Peak intensities of fragment ions in MS/MS spectra were also correlated with structural features and fragmentation preferences of the investigated saponins. In general, the formation of fragments occurred predominantly by cleavages of glycosidic bonds in the positive mode (Figure 12), which was applied to identify the structure of saponins. Interpretation of fragment ions of MS/MS spectra provided the key information for the structural elucidation of saponins as exemplified in Figure 12. 

Fragmentation of the ion at *m/z* 1243.5 (sulfated saponin) under collisionally activated dissociation (CAD) conditions is shown in Figure 12. Full and dotted arrows show the two main fragmentation pathways in this saponin. The peak at *m/z* 507 corresponds to both the aglycone and the key diagnostic fragment of sugar moiety. 

The most abundant peaks were detected at *m/z* 1123 [M + Na − 120 (sulfate)]^+^, 639 [M + Na − 120 − 484 (aglycone)]^+^ and 507 [M + Na − 120 − 484 − 132]^+^. In addition, the peaks observed at *m/z* 1225.5 and 1199.5 were generated by the losses of H_2_O and CO_2_ from their respective parent ion.

The most intensive peak was observed at *m/z* 593 stemming from a cross-ring cleavage. The observed fragments are consistent with the structure of the Holothurin A proposed by Van Dyck *et al.* [50]. This ESI-MS/MS analysis confirmed the MALDI data on the ion at *m/z* 1243.5. The full analysis can be seen in Supplementary Figure S1.

ESI-MS was applied to distinguish the isomeric saponins by Song *et al.* [100]. Isomers of saponins were also identified using tandem mass spectrometry combined with electrospray ionization (ESI-MS/MS) following HPCPC separation. MS/MS spectra of these ions gave detailed structural information and enabled differentiation of the isomeric saponins. The results are exemplified in the following figures. The analyses applied on the ion at *m/z* 1303.6 (non-sulfated saponins), which was obtained from Fractions 15, 14 and 12, are shown in Figure 3A–C. The main fragmentation patterns observed for this isomeric compound are shown with full and dotted arrows.

The structures of six isomeric saponins were ascribed to the ions detected at *m/z* 1303.6 (Figure 3A–C). These isomers have at least three different aglycone structures with *m/z* 468, 484 and 500 and contain five different monosaccharaide residues. These figures illustrate different isomers of ions detected at *m/z* 1303. For instance, Figure 3A (Fraction 15) shows the stepwise structure elucidation of Holothurinoside A. The consecutive losses of MeGlc, Glc, Qui, Glc, and Xyl units generate signals detected at *m/z* 1127.5, 965.5, 819.3, 657.2 and 507.2, respectively, which correspond to Holothurinoside A [5,62,69,88]. As can be seen in Figure 3A, this saponin fraction is quite pure.

In one of these isomers (Figure 3B), the consecutive losses of aglycone, Glc, Xyl, Qui and Glc units provided signals detected at *m/z* 819, 657, 507, 361 and 199, respectively, further confirming that the fragment ions unambiguously originate from sodium-cationized Holothurinoside A. In addition (*m/z* 1303.6), the precursor ion sequentially lost MeGlc (*m/z* 1127.5), Glc (*m/z* 965.5), Glc (*m/z* 803.4), Qui (*m/z* 657.2) and Xyl (*m/z* 507.2) (Figure 3B) thereby indicating the structure of another isomer of this molecule. The characteristic peak observed at *m/z* 507.2 generated by tandem MS was identified either as a sodiated MeGlc-Glc-Qui residue or sodiated aglycone residue. The ion at *m/z* 803 resulted from the loss of aglycone from the parent ion at *m/z* 1303.6, which is the fragment ion corresponding to the complete saccharide chain, which subsequently (Figure 3B) produces the ions at *m/z* 657 and ion at *m/z* 507 by the losses of Qui and Xyl residues. Moreover, the ions (*m/z* 507) further fragmented to form ions of the same *m/z* value at *m/z* 361 and *m/z* 199 or 185. The observation of ions at *m/z* 507 and 657 further supports the above conclusion. The ions detected at *m/z* 1285.5 and 1241.6 correspond to the losses of H_2_O and H_2_O + CO_2_, respectively. These two fragments correspond to the sequential losses of water and carbon dioxide. It is notable that the configurations of all the sugars in all previously known sea cucumber triterpene glycosides are d-configurations. 

A similar analysis was carried out (Figure 3C) on the ion at *m/z* 1303.6 of Fraction 12. As can be seen in the figure, the spectrum has a different fragmentation pattern compared to the spectra in Figure 3A,B even though they have the same *m/z* value. In one of the isomers, the consecutive losses of aglycone, Glc, Xyl, Glc and MeGlc units generated signals detected at *m/z* 835.2, 673.2, 523.1, 361.1 and 185, respectively, further confirming the structure of one of the isomeric compounds (Figure 3C and Figure 5). The full analysis can be seen in the Supplementary Figure S2 (Fraction 12).

Further, the cleavage of the C_2_ ion at *m/z* 673, 643, 629, 601, 583, 541 and 523 (Figure 3C) produced the ion at *m/z* 613, 583, 569, 541, 523, 481 and 463, respectively, through the loss of C_2_H_4_O_2_ (60 Da) which indicates an α 1–4-linked glycosidic bond in the α-chain, which is in agreement with a previous study [100]. This observation is consistent with the fragmentation rules for ions of 1–4-linked disaccharides.

The MS/MS spectra show the presence of three different aglycone structures, namely ions detected at *m/z* 835.2, 819.2 and 803.4 by the losses of aglycone moieties. This analysis reveal the presence of at least six different isomers with different aglycones and sugar moieties, as the MS/MS spectra generate both key diagnostic fragments at *m/z* 507 and 523. These isomers are composed of five monosaccharaides including MeGlc-Glc-Qui (Glc)-Xyl-MeGlc (Glc or Qui). The proposed structures are shown in Figure 5 and correspond to Holothurinoside A_1_ and Holothurinoside A, and four novel saponins [5,69,88]. We propose to call these molecules Holothurinosides S, Q, R and R_1_, respectively. 

It should be noted that both major fragment ions (507 and 523) can correspond to partial glycoside compositions or aglycone moieties, further supporting the presence of isomeric saponins. The predominant fragment ion at *m/z* 507 results from the sodium adduct ion of the [MeGlc-Glc-Qui + Na] side chain or the aglycone. Similarly, the abundant fragment ion at *m/z* 523 arises from the sodium adduct ion of the [MeGlc-Glc-Glc + Na]^+^ side chain or the aglycone. Since the masses of sodiated aglycones are identical with their relative partial sugar residues, namely [MeGlc-Glc-Qui + Na]^+^ and [MeGlc-Glc-Glc + Na]^+^, the ions at *m/z* 507.2 and 523.2, respectively, correspond to both sugar residues and their aglycones. When the decomposition of the parent ion (*m/z* 1303.6) is triggered by the losses of sugar residues, as an exemplified by the black and pink dotted arrows in Figure 3A–C, the ions at *m/z* 507.2 and 523.2 correspond to the aglycone moieties. Alternatively, the fragmentation of the parent ion can proceed by the losses of all five sugar residues, which generates ions at *m/z* 507.2 and 523.2, which correspond to the aglycone moieties. Similar conclusions were drawn by Van Dyck *et al.* (2009) [69] for triterpene glycosides. Losses of H_2_O and CO_2_ or their combination result from cleavage at the glycosidic linkages as noted by Waller and Yamasaki [3].

Different fractions of the HPCPC separation were compared to show the presence of one aglycone (Figure 3A), the presence of two different aglycones (Figure 3B) and the presence of three different aglycones (Figure 3C) indicating that the HPCPC allowed the separation of the isomers.

On comparison of the MS/MS spectra of 1303.6 and 1243.5 (Figure 3 and Figure 12), it is notable that the *m/z* 523 fragment (aglycone loss) of the [M + Na]^+^ ions was only observed with 1303.6, which corresponds to the presence of a new aglycone unit at *m/z* 500 (sodiated 523). Individual patterns were detected from sulfated and non-sulfated saponins as indicated in Holothurin A and Holothurinoside A as representative examples. This sequential decomposition confirms the proposed Holothurin A and Holothurinoside A structures.

Another typical chemical structure elucidation of isomeric saponins by tandem MS is exemplified in Figure 6. This spectrum shows the ion signature of the sample under tandem MS from the ion detected at *m/z* 1287.6. Tandem MS analyses revealed the presence of two different aglycones with *m/z* values of 484 and 468, confirming the presence of chemical isomeric structures. The same fragmentation behaviors have been observed from the positive ESI-MS/MS spectra of saponins with *m/z* 1303. The structures of aglycones are identical with those reported for the ion at *m/z* 1303. The possible fragmentation pathways were shown using full and dotted arrows. The losses of aglycone moieties (Figure 6) generated ions at *m/z* 819.3 and 803.4, which correspond to the complete sugar components. The successive losses of aglycone, Glc or MeGlc, Xyl, Qui and MeGlc yielded to ion fragments at *m/z* 819, 657 or 643, 507, 361 and 185, respectively.

The decomposition of the parent ion can also be triggered by the loss of a sugar moiety, namely MeGlc, Glc, Qui, Qui or Glc and Xyl, followed by the aglycone, which generates daughter ions at *m/z* 1111.5, 949.5, 803.4, 657.2 or 643.2 and 507.2. It is clear that the ion at *m/z* 507 is the most abundant fragment ion and is the signature of the sodiated aglycone and/or the key sugar component. The losses of water (−18 Da) and/or carbon dioxide (−44 Da) are observed from the spectrum, and some of the peaks are also designated to those molecules.

This analysis revealed the presence of at least five different isomers with different aglycones and sugar moieties. These isomers contain some identical aglycone structures with those identified in the ion at 1303 (Figure 5). These isomers are also pentaglycosidic saponins. The proposed structures are shown in Figure 7, which correspond to Holothurinoside E_1_, Holothurinoside E, 17-dehydroxy-holothurinoside A and two novel saponins (the first and fourth compounds). We propose to name these molecules Holothurinosides O and P, respectively. 

The data indicate that the terminal sugar is preferentially lost first in glycosidic bond cleavages. Since Holothurinosides A and E contain the same terminal sugar units in their sugar residue, they yield the ions with the same *m/z* value (*m/z* 507).

## 3. Experimental Section

### 3.1. Sea Cucumber Sample

Twenty sea cucumber samples of *Holothuria lessoni* Massin *et al.* 2009, commonly known as Golden sandfish were collected off Lizard Island (latitude 14°41′29.46″ S; longitude 145°26′23.33″ E), Queensland, Australia in September 2010. The viscera (all internal organs) were separated from the body wall and kept separately in zip-lock plastic bags which were snap-frozen, then transferred to the laboratory and kept at −20 °C until use. 

### 3.2. Extraction Protocol

The debris and sand particles were separated from the viscera (all internal organs) manually and the visceral mass was freeze-dried (VirTis, BenchTop K, New York, NY, USA). The dried specimens were then pulverized to a fine powder using liquid nitrogen and a mortar and pestle. 

All aqueous solutions were prepared with ultrapure water generated by a Milli-Q systems (18.2 MΩ, Millipore, Bedford, MA, USA). All organic solvents were purchased from Merck (Darmstadt, Germany) except when the supplier was mentioned, and were either of HPLC grade or the highest degree of purity. 

#### Extraction of Saponins

The extraction and purification procedures were adapted from Campagnuolo *et al.* [34], Van Dyck *et al.* [69], Garneau *et al.* [102] and Grassia *et al.* [103]. The pulverized viscera sample (40 g) was extracted four times with 70% ethanol (EtOH) (400 mL) followed by filtration through Whatman filter paper (No.1, Millipore, Bedford, MA, USA) at room temperature. The extract was concentrated under reduced pressure at 30 °C using a rotary evaporator (Büchi AG, Flawil, Switzerland) to remove the ethanol, and the residual sample was freeze-dried to remove water (VirTis, BenchTop K, New York, NY, USA). The dried residue was successively extracted using a modified Kupchan partition procedure [104]: The dried extract (15 g) was dissolved in 90% aqueous methanol (MeOH) (any remaining solid residue was removed by filtration), and partitioned against 400 mL of *n*-hexane (v/v) twice. The water content of the hydromethanolic phase was then adjusted to 20% (v/v) and then to 40% (v/v) and the solutions partitioned against CH_2_Cl_2_ (450 mL) and CHCl_3_ (350 mL), respectively. In the next step, the hydromethanolic phase was concentrated to dryness using a rotary evaporator and freeze-drier. The dry powder was solubilized in 10 mL of MilliQ water (the aqueous extract) in order to undergo chromatographic purification.

### 3.3. Purification of the Extract

A solution of the aqueous extract was then subjected to a prewashed Amberlite^®^ XAD-4 column (250 g XAD-4 resin 20–60 mesh; Sigma-Aldrich, MO, USA; 4 × 30 cm column) chromatography. After washing the column extensively with water (1 L), the saponins were eluted sequentially with MeOH (450 mL) and acetone (350 mL) and water (250 mL). The eluates (methanolic, acetone and water fractions) were then concentrated, dried, and redissolved in 5 mL of MilliQ water. Finally, the aqueous extract was partitioned with 5 mL isobutanol (v/v). The isobutanolic saponin-enriched fraction was either stored for subsequent mass spectrometry analyses or concentrated to dryness and the components of the extract were further examined by HPCPC and RP-HPLC. The profile of fractions was also monitored by Thin Layer Chromatography (TLC) using the lower phase of CHCl_3_/MeOH/H_2_O (7:13:8 v/v/v) solvent system.

### 3.4. Thin Layer Chromatography (TLC)

Samples were dissolved in 90% or 50% aqueous MeOH and 10 microliters were loaded onto silica gel 60 F_254_ aluminum sheets (Merck #1.05554.0001) and developed with the lower phase of CHCl_3_/MeOH/H_2_O (7:13:8) biphasic solvent system. The profile of separated compounds on the TLC plate was visualized by UV light and by spraying with a 15% sulfuric acid in EtOH solution and heating for 15 min at 110 °C until maroon-dark purple spots developed.

### 3.5. High Performance Centrifugal Partition Chromatography (HPCPC or CPC)

The solvent system containing CHCl_3_/MeOH/H_2_O–0.1% HCO_2_H (7:13:8) was mixed vigorously using a separating funnel and allowed to reach hydrostatic equilibration. Following the separation of the two-immiscible phase solvent systems, both phases were degassed using a sonicator-degasser (Soniclean Pty Ltd., Adelaide, SA, Australia). Then the rotor column of HPCPC™, CPC240 (Ever Seiko Corporation, Tokyo, Japan) was filled with the liquid stationary phase at a flow rate of 5 mL/min by Dual Pump model 214 (Tokyo, Japan).

The CPC was loaded with the aqueous upper phase of the solvent system in the descending mode at a flow rate of 5 mL/min with a revolution speed of 300 rpm. The lower mobile phase was pumped in the descending mode at a flow rate of 1.2 mL/min with a rotation speed of 900 rpm within 2 h. One hundred and twenty milligrams of isobutanol-enriched saponin mixture was dissolved in 10 mL of the upper phase and lower phase in a ratio of 1:1 and injected to the machine from the head-end direction (descending mode) following hydrostatic equilibration of the two phases indicated by a clear mobile phase eluting at the tail outlet. This indicated that elution of the stationary phase had stopped and the back pressure was constant. The chromatogram was developed at 254 nm for 3.0 h at 1.2 mL/min and 900 rpm using the Variable Wavelength UV-VIS Detector S-3702 (Soma optics Ltd., Tokyo, Japan) and chart recorder (Ross Recorders, Model 202, Topac Inc., Cohasset, MA, USA). The fractions were collected in 3 mL/tubes using a Fraction collector. The elution of the sample with the lower organic phase proceeded to remove the compounds with low polarity from the sample, within 200 mL of which several peaks were eluted. At this point (Fraction 54), the elution mode was switched to ascending mode and the aqueous upper phase was pumped at the same flow rate for 3.0 h. Recovery of saponins was achieved by changing the elution mode to the aqueous phase which allowed the elution of the remaining compounds with high polarity in the stationary phase. A few minor peaks were also monitored. Fractions were analyzed by TLC using the lower phase of CHCl_3_/MeOH/H_2_O (7:13:8) as the developing system. The monitoring of the fractions is necessary, as most of the saponins were not detected by UV due to the lack of a chromophore structure. Fractions were concentrated with nitrogen gas.

### 3.6. Mass Spectrometry

The isobutanol saponin-enriched fractions and the resultant HPCPC purified polar samples were further analyzed by MALDI and ESI MS to elucidate and characterize the molecular structures of compounds. 

#### 3.6.1. MALDI-MS

MALDI analysis was performed on a Bruker Autoflex III Smartbeam (Bruker Daltonik, Bremen, Germany). All MALDI MS equipment, software and consumables were from Bruker Daltonics (Bremen, Germany). The laser (355 nm) had a repetition rate of 200 Hz and operated in the positive reflectron ion mode for MS data over the mass range of 400 to 2200 Da under the control of the FlexControl and FlexAnalysis software (V 3.3 build 108, Bruker Daltonik, Bremen, Germany). External calibration was performed using PEG. MS spectra were processed in FlexAnalysis (version 3.3, Bruker Daltonik, Bremen, Germany). MALDI MS/MS spectra were obtained using the LIFT mode of the Bruker Autoflex III with the aid of CID. The isolated ions were submitted to collision against argon in the collision cell to collisionally activate and fragment, and afford intense product ion signals. For MALDI, a laser energy was used that provided both good signal levels and mass resolution, the laser energy for MS/MS analysis was generally 25% higher than for MS analysis.

The samples were placed onto a MALDI stainless steel MPT AnchorChip TM 600/384 target plate. Alpha-cyano-4-hydroxycinnamic acid (CHCA) in acetone/ iso-propanol in ratio of 2:1 (15 mg/mL) was used as a matrix to produce gas-phase ions. The matrix solution (1 μL) was spotted onto the MALDI target plate and air-dried. Subsequently 1μL of sample was added to the matrix crystals and air-dried. Finally, 1 μL of NaI (Sigma-Aldrich #383112, St Louis, MO, USA) solution (2 mg/mL in acetonitrile) was applied onto the sample spots. The samples were mixed on the probe surface and dried prior to analysis. 

#### 3.6.2. ESI-MS

The ESI mass spectra were obtained with a Waters Synapt HDMS (Waters, Manchester, UK). Mass spectra were obtained in the positive ion mode with a capillary voltage of 3.0 kV and a sampling cone voltage of 100 V.

The other conditions were as follows: extraction cone voltage, 4.0 V; ion source temperature, 80 °C; desolvation temperature, 350 °C; desolvation gas flow rate, 500 L/h. Data acquisition was carried out using Waters MassLynx (V4.1, Waters Corporation, Milford, CT, USA). Positive ion mass spectra were acquired in the V resolution mode over a mass range of 100–2000 *m/z* using continuum mode acquisition. Mass calibration was performed by infusing sodium iodide solution (2 μg/μL, 1:1 (v/v) water/isopropanol). For accurate mass analysis a lock mass signal from the sodium attached molecular ion of Raffinose (*m/z* 527.1588) was used.

MS/MS spectra were obtained by mass selection of the ion of interest using the quadrupole, fragmentation in the trap cell where argon was used as collision gas. Typical collision energy (Trap) was 50.0 V. Samples were infused at a flow rate of 5 μL/min, if dilution of the sample was required then acetonitrile was used [100]. Chemical structures were determined from fragmentation schemes calculated on tandem mass spectra and from the literature.

## 4. Conclusions

The extract of the viscera of sea cucumber *H*. *lessoni* was processed by applying HPCPC to purify the saponin mixture and to isolate saponin congeners and isomeric saponins. The tandem MS approach enabled us to determine the structure of a range of saponins. The purity of HPCPC fractions allowed mass spectrometry analyses to reveal the structure of isomeric compounds containing different aglycones and/or sugar residues. Several novel saponins, along with known compounds, were identified from the viscera of sea cucumber. 

This study is the first on saponins from the viscera of sea cucumbers. Our results to date highlight that there are a larger number of novel saponins in the viscera compared to the body wall (data not shown) indicating the viscera as a major source of these compounds. This paper is the first not only to report the presence of several novel saponins in the viscera of *H*. *lessoni* but also to indicate the highest number of saponin congeners detected in the viscera of any sea cucumber species. The mass of reported saponins for this species ranged from 460 Da to 1600 Da. So far we have identified more than ten aglycone structures in this species. Evidence from MALDI-MS suggested that the most intensive saponin ion was *m/z* 1243.5, a major component which seemed to correspond to Holothurin A. However, in the tandem MS, the most abundant ions are generally attributed to the loss of aglycones and/or both key diagnostic sugar moieties (507 and 523). Our results also showed that the incidence of the cross-ring cleavages was higher in the sulfated compounds compared to non-sulfated glycosides. It can be concluded that the presence of a sulfate group in the sugar moiety of saponins made them more vulnerable to cross-ring cleavages.

At the moment, MS is one of the most sensitive techniques of molecular analysis to determine saponin structures. This methodology of molecular structure identification using fragmentation patterns acquired from MS/MS measurements helps to propose and identify the structure of saponins. It was found that under CID some of the identified saponins had the same ion fingerprints for their aglycone units, yielding the same *m/z* daughter ions. Some of these saponins were easily characterized based on MS/MS measurement since their CID spectra contained the key diagnostic signals at *m/z* 507 and 523, corresponding to the oligosaccharide chains [MeGlc-Glc-Qui + Na^+^] and [MeGlc-Glc-Glc + Na^+^], respectively. The simultaneous loss of two sugar units indicated characteristics of a branched sugar chain. This methodology also permitted the structural elucidation of isomers.

Sea cucumbers have developed a chemical defense against potential predators based upon saponins. Our finding indicates that the viscera are rich in saponins, in both diversity and quantity, and that these saponins are apparently more localized in the viscera than in the body wall.

The chromatography techniques used in this study were able to for the first time, separate high purity saponins from sea cucumber, highlight the diversity of saponin congeners, and stress the unique profile of saponins for this species. MALDI and ESI-MS proved to be sensitive, ultra-high-throughput methodologies to identify these secondary metabolites in a complex mixture. Therefore, mass spectrometry has become the preferred techniques for analysis of saponins, as both ESI-MS and the MALDI-MS spectra provide remarkable structural information. However, the MALDI data is simpler to interpret compared to ESI-MS data due to the singly charged ions. This ancient creature with a long evolutionary history is a unique source of high-value novel compounds.

This manuscript describes the structure elucidation of seven novel compounds; Holothurinoside O, Holothurinoside P, Holothurinoside Q, Holothurinoside R, Holothurinoside R_1_, Holothurinoside S and Holothurinoside T in addition to six known compounds, including Holothurin A, Holothurinoside A, Holothurinoside A_1_, Holothurinoside E, Holothurinoside E_1_ and 17-dehydroxy-holothurinoside A. 

In conclusion, our findings show that the viscera of *H*. *lessoni* contain numerous unique and novel saponins with a high range of structural diversity, including both sulfated and non-sulfated congeners, and with different aglycone and sugar moieties. Furthermore, the tremendous range of structural biodiversity of this class of natural metabolites, which enables them to present in a remarkable functional diversity, is potentially an important source for the discovery of high-value compounds for biotechnological applications.

## Supplementary Files

Supplementary File 1 Supplementary Information (PDF, 183 KB)
