# Supplementary material for: Discovery of Novel Saponins from the Viscera of the Sea Cucumber Holothuria lessoni
Source: Mar Drugs. 2014 May 9;12(5):2633–67. doi: 10.3390/md12052633 (PMC4052309; doi:10.3390/md12052633)
Supplement: Supplementary File 1 — Supplementary Information (PDF, 183 KB) [file marinedrugs-12-02633-s001.pdf]

Supplementary Information

Figure S1. This picture shows the possible ion fragmentations and proposed structures for the Holothurin A, the precursor ion at *m/z* 1243.5.

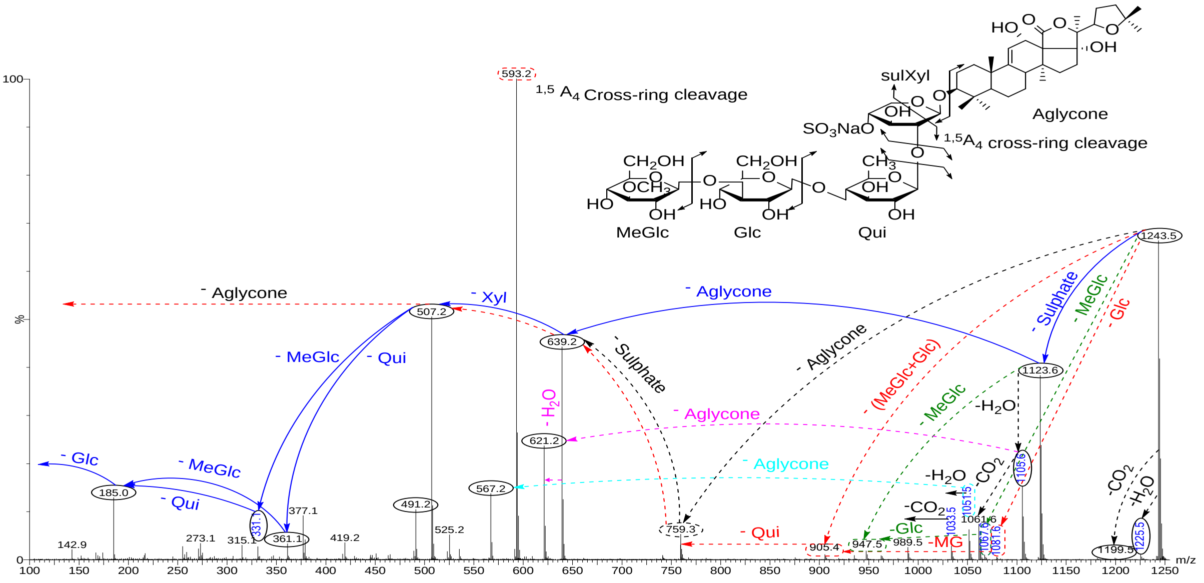

Figure S2. This picture shows the possible ion fragmentations and proposed structures for the isomeric saponins of the precursor ion at *m/z* 1303.6 from fraction 12.

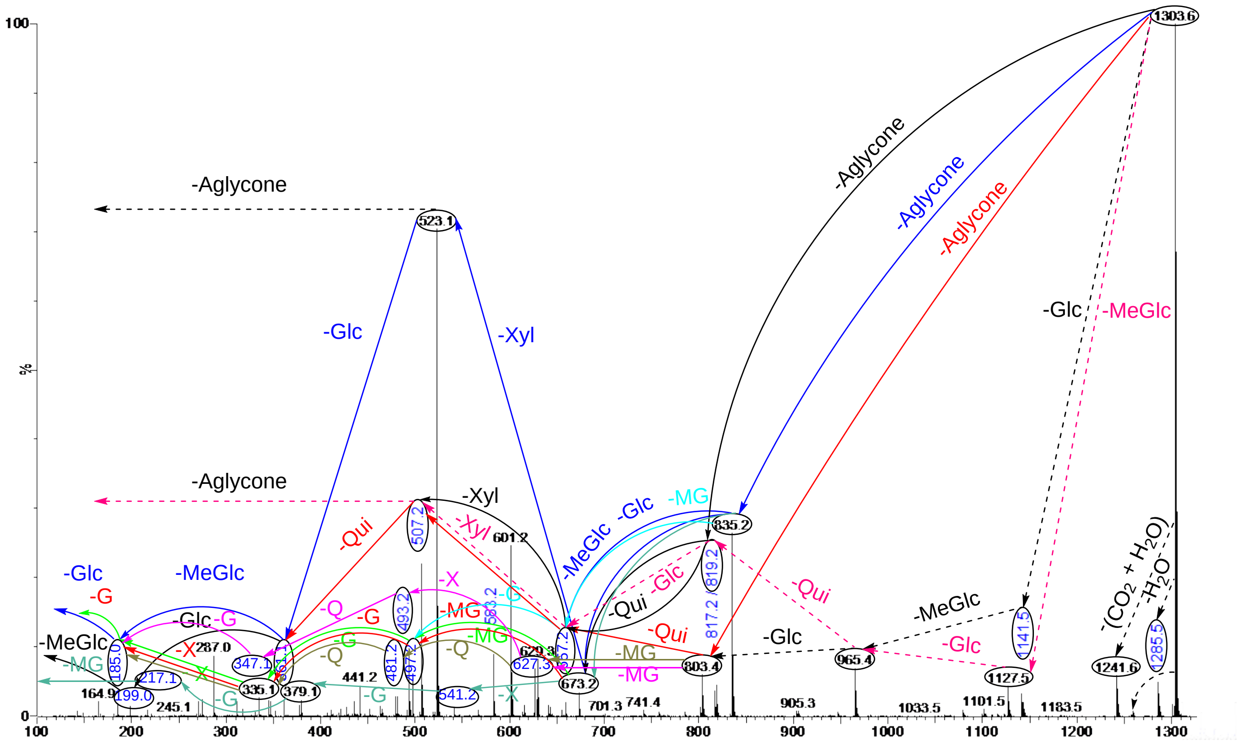

Abbreviations; MG = MeGlc, G = Glc, Q = Qui, X = Xyl.

© 2014 by the authors; licensee MDPI, Basel, Switzerland. This article is an open access article distributed under the terms and conditions of the Creative Commons Attribution license (http://creativecommons.org/licenses/by/3.0/).
